# Unraveling the promise and challenges of biomarkers in pediatric sepsis: a commentary on risk estimation of organ dysfunction and immune dysregulation

**DOI:** 10.1038/s41390-025-03983-5

**Published:** 2025-03-17

**Authors:** Kenneth E. Remy, Niranjan Kissoon

**Affiliations:** 1 Department of Medicine, Case Western University School of Medicine, University Hospitals of Cleveland, Cleveland, OH USA; 2 Department of Pediatrics, Case Western University School of Medicine, Rainbow Babies and Children’s Hospital, Cleveland, OH USA; 3 Department of Pediatrics, University of British Columbia, Vancouver, BC Canada

## Introduction

Sepsis is a life-threatening syndrome resulting from a dysregulated immune response to infection, leading to systemic inflammation, organ dysfunction, and immune dysregulation. Timely and accurate identification of sepsis is paramount for guiding treatment strategies and improving clinical outcomes, particularly in pediatrics, where unique physiological factors complicate diagnosis and management.

In recent years, biomarkers have gained significant attention as potential tools for risk stratification in pediatric sepsis, offering insights into the underlying pathophysiology and facilitating personalized therapeutic approaches. Biomarkers provide quantifiable measures of disease activity, organ dysfunction, and immune response dysregulation. They have the potential to augment clinical assessment, refine prognostication, and tailor therapeutic interventions. For instance, biomarkers of organ dysfunction, such as cardiac troponins and renal markers like neutrophil gelatinase-associated lipocalin (NGAL), elucidate the extent and progression of organ injury, enabling early and targeted interventions. Similarly, biomarkers of immune dysregulation, including cytokines and markers of immune cell exhaustion, reveal insights into the host response to infection, helping to identify patients at risk of secondary complications or immunosuppression.

Jariyasakoolroj and colleagues comprehensively reviewed biomarkers associated with immune dysregulation and organ dysfunction in this issue, underscoring their utility in pediatric sepsis.^1^ Strategies that focus on the host response has been suggested as the enabler of personalized medicine almost a decade ago.^2^ While the potential of biomarkers is undeniable, challenges remain in their interpretation and integration into clinical practice due to heterogeneity in sepsis presentations, resource limitations, and the complexity of pediatric immune responses. These barriers must be addressed to unlock the full potential of biomarkers in advancing pediatric sepsis care.

## Why are biomarkers helpful?

Biomarkers offer a window into the molecular and physiological pathways that underpin disease processes in pediatric sepsis.^3–5^ They enable clinicians to identify high-risk patients, monitor disease progression, and implement precision-based interventions. However, significant variability in sepsis pathophysiology—owing to differences in age, comorbidities, causative organisms, and timing of clinical presentation—renders biomarker interpretation challenging. For example, elevated serum lactate, a marker of tissue hypoxia, can signal severe disease and guide early interventions such as fluid resuscitation. However, in pediatric populations, lactate levels are influenced by age-related metabolic differences and underlying conditions, complicating their use as a definitive marker of sepsis. Similarly, comorbidities and concurrent therapies, such as corticosteroids, may alter biomarker profiles, necessitating careful interpretation.

Predictive biomarkers hold promise for guiding therapeutic decisions by identifying patients likely to benefit from specific interventions.^6^ By identifying high-risk patients early in the disease course, biomarkers may enable targeted interventions aimed at modulating the immune response and mitigating organ dysfunction. For instance, ongoing research by Hall et al. utilizes ex vivo stimulation of whole blood with lipopolysaccharides (LPS) to stratify pediatric sepsis patients for granulocyte-macrophage colony-stimulating factor (GM-CSF) therapy (clinicaltrials.gov NCT05266001). Such approaches exemplify the potential of biomarkers to inform precision medicine.

## Biomarkers in organ dysfunction and sepsis

Mortality rates in pediatric sepsis are relatively low compared to adults, necessitating a focus on morbidity indicators such as organ dysfunction. Biomarkers of organ dysfunction have emerged as key tools for early recognition and risk stratification. Examples include: (1) *Cardiac biomarkers:* Troponins and brain natriuretic peptide (BNP) indicate myocardial injury and dysfunction, (2) *Renal biomarkers:* NGAL and kidney injury molecule-1 (KIM-1) provide early indicators of acute kidney injury, (3) *Pulmonary biomarkers:* Surfactant protein D (SP-D) and soluble receptor for advanced glycation end products (sRAGE) are linked to acute respiratory distress syndrome (ARDS), and (4) *Neurological biomarkers:* S100B protein and neuron-specific enolase (NSE) signal neuronal injury, potentially identifying patients at risk of sepsis-associated encephalopathy.^1,6,7^

Likewise, biomarkers of immune dysregulation are essential for understanding maladaptive immune responses in pediatric sepsis, which drive inflammation, immunosuppression, and secondary infections. They aid in assessing immune status and guiding therapeutic interventions. Jariyasakoolroj et al. categorize these biomarkers as inflammatory (e.g., CRP, PCT, IL-6, linked to severity and outcomes), anti-inflammatory (e.g., IL-10, TGF-β, indicating immunosuppression), cellular (e.g., PD-1, CTLA-4, reflecting immune exhaustion), and endothelial (e.g., Ang-2, vWF, sICAM-1, associated with vascular injury).^1^ Despite their potential, these biomarkers have often failed to achieve widespread clinical adoption due to their lack of specificity, inability to differentiate sepsis from non-infectious inflammatory conditions, and variability across populations and clinical contexts.

Future efforts to improve biomarker utility should focus on enhancing their accuracy and precision through multiparametric approaches, integrating them into composite panels that account for dynamic and temporal changes in sepsis. Standardizing assay methods, validating biomarkers across diverse populations, and incorporating advanced computational tools such as machine learning to analyze biomarker interactions could also refine their clinical relevance. By linking biomarkers more closely to actionable pathways and individual patient profiles, future research can better align these tools with the goals of precision medicine in sepsis care.^3–5,7^

## Integration with the new Phoenix criteria for sepsis diagnosis

Biomarkers show promise for risk estimation but face challenges with cost, availability, and interpretation. The Phoenix Criteria offer a robust system for identifying pediatric sepsis and septic shock through a composite score incorporating dysfunctions in respiratory, cardiovascular, coagulation, and neurological systems. Biomarkers like lactate and coagulation factors play a critical role in enhancing diagnostic and prognostic accuracy.^8,9^ However, currently used nonspecific markers, such as CRP and PCT, lack the precision to capture the underlying biology or mechanisms of disease severity, nor provide insights into mechanisms explaining differences in disease severity. The Phoenix Sepsis Criteria enhance their utility by combining clinical data with biomarkers like lactate and coagulation markers, improving the diagnosis and stratification of pediatric sepsis.

Incorporating novel, more specific biomarkers like dipeptidyl peptidase 3 (DPP3) and functional cytokine responses into frameworks like the Phoenix Score can significantly improve the early identification and management of pediatric sepsis, enabling a more precise and effective approach to care.^8,9^ Integrating novel and more specific biomarkers into the Phoenix Sepsis Score enhances its predictive capability.

## Challenges faced with biomarker integration

While these biomarkers hold promise, we are in the infancy of understanding the complexities on how to integrate these biomarkers as ‘mono’ markers into a more multidimensional and dynamic platform that includes clinical and laboratory variables to characterize stages of sepsis to guide clinician ‘action.’ Despite their promise, biomarkers are not yet seamlessly integrated into routine pediatric sepsis management. Validation is hindered by patient heterogeneity, which complicates the identification of universal biomarkers. Serial measurements, often necessary to capture the dynamic nature of sepsis, may not be feasible in resource-limited settings. Additionally, the effects of comorbidities and concurrent therapies, such as immunomodulatory agents, further confound biomarker interpretation. Comorbidities and medications, like corticosteroids or biologics, can alter baseline levels and affect interpretation, complicating risk stratification.^10,11^ Moreover, The dynamic nature of sepsis requires serial biomarker measurements, which may not always be feasible. Comorbidities like chronic kidney disease, autoimmune disorders, and medications can alter baseline levels, complicating risk stratification. Immunomodulatory therapies, such as corticosteroids or biologics, also impact biomarker kinetics, necessitating careful interpretation.

Developing a multidimensional platform that integrates biomarkers with clinical and laboratory variables is critical for addressing these challenges. Such platforms could enable stage-specific characterization of sepsis and guide timely clinical actions.

## Next steps

To realize the potential of biomarkers in pediatric sepsis, collaborative efforts are essential. Multicenter research consortia and prospective cohort studies can validate biomarker panels across diverse populations, including low- and middle-income countries where sepsis disproportionately impacts children. Emerging technologies, such as multiplex assays and point-of-care testing, could improve biomarker accessibility in resource-limited settings.^12^ Sadly, biomarker use is limited by a lack of research and validation in diverse populations. Multi-omic studies could offer insights but are costly and underfunded. Reproducibility and generalizability across healthcare settings are also challenges. Incorporating new biomarkers into the Phoenix Criteria and using AI and machine learning may improve sepsis care, but it is too early to assess their impact.

Future research should focus on identifying novel biomarkers that reflect underlying biology, elucidating mechanisms of disease, and incorporating validated biomarkers into decision-making algorithms. Advances in artificial intelligence and machine learning may further enhance biomarker integration by uncovering patterns in large datasets. Ultimately, biomarkers must transcend their role as diagnostic adjuncts to become actionable tools that guide personalized interventions and improve outcomes. Furthermore, advancements in technology, such as point-of-care testing and multiplex assays, may enhance the feasibility and accessibility of biomarker-based approaches in resource-limited settings.

Biomarkers must serve to reflect the underlying biology that can be modulated to improve outcomes. Without biologic understanding, improved interpretation, and the ability to utilize biomarkers to guide therapy, we will be faced with ‘just more tests’ to order without meaningful purpose.
